# Wrong rulings come from wrong science

**DOI:** 10.1186/s40834-024-00281-z

**Published:** 2024-04-15

**Authors:** Anita Nelson, Margaret English

**Affiliations:** 1 Los Angeles, CA USA; 2 Department of OBGYN, Harbor-UCLA Medical Center, Torrance, CA USA

## Introduction

The problem is, when you start with a wrong premise, your conclusions will almost certainly be wrong. Based on a series of increasingly incorrect decisions, the Supreme Court in Alabama felt confident in stating at the beginning of its analysis of the “IVF case” (i.e. *LePage v Center for Reproductive Medicine*) that “all parties to these cases, like all members of the Court, agree that an unborn child is a genetically unique human being whose life begins at fertilization and ends at death.” Given this premise, the only question the Court felt necessary to address was if an exception to the Wrongful Death of a Minor Act could exist for “unborn children” who are not physically located in utero. The judge ruled that the Act applies to all unborn children, regardless of their location.

## Definitional issues

Although the US Supreme Court has ruled that scientific evidence is not a basis for judicial review, thinking people might want to consider the basis, logic, and clinical implications of such a decision.

The first issue is the problematic premise that a frozen embryo, or “extrauterine child” as Alabama coins them in this decision, is life. Life in its simple scientific definition (fond memories of high school biology, anyone? ) is a condition that separates that which can independently grow, develop, and reproduce from inorganic matter. Simply put, a frozen embryo is not life. This should not be controversial— it is clear, to these authors at the very least, that an embryo does not have any of these qualities of life outside of a uterus.

Putting that aside, a similar but distinct issue is the claim that “life” starts with fertilization. This case relies on legislation called the Wrongful Death of a Minor Act, which was first passed in 1872 Alabama. In his *LePage* ruling, the judge carefully listed factors that were considered when interpreting and applying this historic piece of legislation in his contemporary decision. Among those factors were the prevailing definitions and original public meanings at the time. As proof that the Act should apply to “unborn babies,” the judge quoted Webster’s dictionary from 1864 that “to be with child (means) to be pregnant.” However, this definition is irrelevant. The fact that humans arise from the union of egg and sperm was not recognized until decades later, after passage of the Act. When this dictionary was published, there was no understanding of human conception. In those days, pregnancy was usually confirmed by quickening in the second trimester. Although the judge in the *LePage* case cited “evidence of original public meaning” as being important in interpreting this piece of legislation, it is *impossible* that this knowledge was part of the intent of the original authors, as “fertilization” was not a meaningful term when the Wrongful Death of a Minor Act was passed.

## Clinical implications

There are even more troubling aspects of this ruling. How can a woman reliably detect death in a conceptus? Most conceptions do not successfully implant, even outside the laboratory setting. When in vivo fertilized eggs do not implant but pass into the menstrual effluent– are they really dead? How can the menstruating person know? Should the woman be held liable for cold heartedly flushing those “unborn babies” down the toilet? To our knowledge, tampon and menstrual cup companies have not been tried in open court for helping dispose of human remains.

A third disturbing consequence of the decision to apply the Wrongful Death of a Minor Act to all unborn children, regardless of their location, can be seen in ectopic pregnancies. With virtually no hope for future development into a viable pregnancy or, as the judge terms them, a “unique being,” are those who save the patient’s life by removing the ectopic pregnancy liable under this Alabama ruling? Are women who choose life-saving interventions endangering their “extrauterine children”?

The rapid actions taken by the Alabama legislature in response to public outcry also shine a light onto a greater motif. When judicial rulings attacked a pronatalist position, politicians worked rapidly to identify an exception to the egregiously misogynous and patently harmful judgement. However, when efforts are made to limit couples’ control over their fertility in other spheres, such as the provision of abortion or contraception, it prompts very slow or no legislative responses. The current fear among reproductive health specialists and advocates for reproductive justice is that, based on the public and legal system’s current misunderstanding of fertilization and the establishment of pregnancy, severe restrictions will be placed on access to contraceptive methods in the name of protecting fertilized “life”.

## New approaches

Attempts have been made to help solve these problems by clarifying the terminology. After fertilization, the conceptus has perhaps been best described as “potential life”. This supports the notion that successful implantation does not predict ultimate successful *extrauterine* life. Even if this scientific definition of life is not universally accepted and people rely on religious teachings, we also must respect that different religions define the onset of life at different times during gestation. Furthermore, adopting one religion’s current definition of life as the universal standard for all citizens violates the first amendment.

We fully acknowledge that the legal system has its own logic and rules. But when those rules so blatantly conflict with reality and cause tangible harm, both physical and psychological, they should be reconsidered. Of course, we also recognize that this commentary is addressing like-minded readers and will likely not reach those most in need of seeing the harms that their decisions are inflicting. However, we do hope some of these thoughts may percolate out to those who may be intellectually curious.

One final question to pose to those who build their arguments on legislation adopted at a time when the issues raised in this case could not even be conceived– would they like to receive medical care that is based on the scientific understanding prevalent at the time the Wrongful Death of a Minor Act was passed? Ask the legal system to catch up.

## Declarations

### Disclaimer

The expressed views of this editorial are the authors’ and not those of any other institution.

